# Association of *NOD2* and *IFNG* single nucleotide polymorphisms with leprosy in the Amazon ethnic admixed population

**DOI:** 10.1371/journal.pntd.0008247

**Published:** 2020-05-20

**Authors:** André Luiz Leturiondo, Ariani Batista Noronha, Carla Yael Ribeiro Mendonça, Cynthia de Oliveira Ferreira, Lucia Elena Alvarado-Arnez, Fernanda Saloum de Neves Manta, Ohanna Cavalcanti de Lima Bezerra, Elizeu Fagundes de Carvalho, Milton Ozório Moraes, Fabíola da Costa Rodrigues, Carolina Talhari

**Affiliations:** 1 Programa de Pós-Graduação em Medicina Tropical, Universidade do Estado do Amazonas, Manaus, Brazil; 2 Laboratório de Biologia Molecular, Fundação Alfredo da Matta, Manaus, Brazil; 3 Coordinación de Investigación, Universidad Franz Tamayo/UNIFRANZ, La Paz, Bolivia; 4 Laboratório de Hanseníase, Instituto Oswaldo Cruz—Fiocruz, Rio de Janeiro, Brazil; 5 Laboratório de Diagnóstico por DNA, Universidade do Estado do Rio de Janeiro, Rio de Janeiro, Brazil; 6 Curso de Medicina, Universidade Nilton Lins, Manaus, Brazil; Lausanne university hospital, SWITZERLAND

## Abstract

Leprosy is a chronic infectious disease, caused by *Mycobacterium leprae*, which affects skin and peripheral nerves. Polymorphisms in genes associated with autophagy, metabolism, innate and adaptive immunity confer susceptibility to leprosy. However, these associations need to be confirmed through independent replication studies in different ethnicities. The population from Amazon state (northern Brazil) is admixed and it contains the highest proportion of Native American genetic ancestry in Brazil. We conducted a case-control study for leprosy in which we tested fourteen previously associated SNPs in key immune response regulating genes: *TLR1* (rs4833095), *NOD2* (rs751271, rs8057341), *TNF* (rs1800629), *IL10* (rs1800871), *CCDC122/LACC1* (rs4942254), *PACRG/PRKN* (rs9356058, rs1040079), *IFNG* (rs2430561), *IL6* (rs2069845), *LRRK2* (rs7298930, rs3761863), *IL23R* (rs76418789) and *TYK2* (rs55882956). Genotyping was carried out by allelic discrimination in 967 controls and 412 leprosy patients. Association with susceptibility was assessed by logistic regression analyses adjusted for the following covariates: gender, age and ancestry. **G**enetic ancestry was similar in case and control groups. Statistically significant results were only found for *IFNG* and *NOD2*. The rs8057341 polymorphism within *NOD2* was identified as significant for the AA genotype (OR = 0.56; 95% CI, 0.37–0.84; P = 0.005) and borderline for the A allele (OR = 0.76; 95% CI, 0.58–1.00; P = 0.053) and carrier (OR = 0.76; 95% CI, 0.58–1.00; P = 0.051). The rs2430561 SNP in *IFNG* was associated with disease susceptibility for the AT genotype (OR = 1.40; 95% CI, 1.06–1.85; P = 0.018) and carrier (OR = 1.44; 95% CI, 1.10–1.88; P = 0.008). We confirmed that *NOD2* and *IFNG* are major players in immunity against *M*.*leprae* in the Amazon ethnic admixed population.

## Author summary

Leprosy is chronic infectious diseases caused by *Mycobacterium leprae* that affect the skin and peripheral nerves. The incidence is still high where approximately 200,000 new cases are diagnosed each year. There is no clear sign for early diagnosis and transmission is likely to occur before treatment, which, irrespective of its success, has not hampered stationary incidence in the past 20 years. Thus, there is pressing need for markers that discriminate exposure, infection and disease in order to better detect leprosy progression, control transmission and prevent disabilities. Here, we investigated whether polymorphisms located in eleven genes are associated to leprosy in a population from Amazon state (northern Brazil) which is admixed and it contains the highest proportion of Native American genetic ancestry in Brazil. We validated *NOD2* and *IFNG* associations with resistance and risk of leprosy, respectively, in the Amazon ethnic admixed population. Genetic patterns of leprosy susceptibility could have an impact on the prognosis of individuals that are more likely to develop the disease (among household contacts, for example). Therefore, this strategy could identify high-risk individuals prone for prophylactic measures such as treatment with single-dose rifampicin and BCG vaccination.

## Introduction

Two hundred thousand new cases of Leprosy are continuously diagnosed every year. The disease is caused by *Mycobacterium leprae* that has a long incubation period, leads to nerve damage and development of physical disabilities [1]. There is no clear sign for early diagnosis and transmission is likely to occur before treatment, which, irrespective of its success, has not hampered stationary incidence in the past 20 years. Thus, there is pressing need for markers that discriminate exposure, infection and disease in order to better detect leprosy progression, control transmission and prevent disabilities. Genetic factors have proved to be key components for leprosy outcome. Studies with monozygotic/dizygotic twins, family, population-based designs, and more recently, genome-wide association studies (GWAS) and whole exome sequencing (WES) have pinpointed single nucleotide polymorphisms (SNPs) in genes that have been consistently replicated in different populations [2–6]. There is evidence that *NOD2*, *LRRK2*, *TLR1*, *TNF*, *IFNG*, *IL10*, *IL23R*, *TYK2* and *PACRG/PRKN* (formerly *PARK2*), which are genes that participate in autophagy and recognition pathways, regulating the host innate immune response are associated with leprosy susceptibility, reaction or its clinical forms [6–18]. However, genetic association of SNPs in major genes that modulate the immune response need independent replication in different ethnic groups to confirm leprosy outcome [19]. As observed in other regions, the Brazilian population from Amazon state is admixed, having three main ancestral contributions: Native Americans (NAM), Europeans (EUR) and Africans (AFR). Of these, the Native American contribution is highest, even within the urban populations of the region [20].

Genetic patterns of leprosy susceptibility could have an impact on the prognosis of individuals that are more likely to develop the disease (among household contacts, for example). Therefore, this strategy could identify high-risk individuals prone for prophylactic measures such as treatment with single-dose rifampicin and BCG vaccination [21–23].

Here, we investigated whether SNPs located in eleven genes: *CCDC122/LACC1*, *IFNG*, *IL6*, *IL10*, *IL23R*, *LRRK2*, *NOD2*, *PACRG/PRKN*, *TLR1*, *TNF* and *TYK2* are associated to leprosy susceptibility in a population in the North of Brazil. To avoid spurious associations due to population stratification, as in the case of admixed populations such as Brazilians, we included genetic ancestry estimates, gender and age, as covariates in the logistic regression analysis.

## Materials and methods

### Ethical statement

This study was approved by the Research and Ethic Committee (N^o^555.620) of the Alfredo da Matta Foundation. All participants signed an informed consent before enrolment. Parents or legal guardians provided consent for participants under the age of 18. This study was performed in accordance with the guidelines strengthening the reporting of genetic association studies (STREGA) [24].

### Design and study population

We performed a case-control study involving individuals from Manaus, a city in the Brazilian state of Amazonas, at the Alfredo da Matta Foundation (FUAM). Patients and controls were recruited from March 2014 to March 2017 by spontaneous demand at FUAM. Patients with leprosy were classified according to the clinical and laboratorial findings (slit skin smears and histopathological examination) and were treated as paucibacillary or multibacillary, according to World Health Organization guidelines. The control group was composed of individuals who lived in the same endemic area of the cases. They were subjected to a dermatoneurological examination, had no suspected leprosy lesions and declared not having contact with leprosy or tuberculosis patients.

### Determination of SNP for genotypic analysis

Several genes have been tested associated in populations although few SNPs were already extensively evaluated and consensus estimates were calculated in meta-analysis (S1 Table). The present study was designed to investigate the association of *TLR1*, *NOD2*, *TNF*, *IL10*, *CCDC122/LACC1*, *PACRG/PRKN*, *IFNG*, *IL6*, *LRRK2*, *IL23R* and *TYK2* genes with leprosy. The SNPs *TLR1* (rs4833095), *NOD2* (rs751271, rs8057341), *TNF* (rs1800629), *IL10* (rs1800871), *CCDC122/LACC1* (rs4942254), *PACRG/PRKN* (rs9356058, rs1040079), *IFNG* (rs2430561) and *IL6* (rs2069845) were selected based on previously published data which showed their association in the Brazilian population to leprosy *per se* [3, 7, 10, 11, 13, 25] or reaction [14]. Based in the previous studies in the Chinese population, we also selected *LRRK2 (*rs7298930, rs3761863) [8], *IL23* (rs76418789) and *TYK2* (rs55882956) [6]. SNPs were included from the promoter, exonic, intronic and chosen based on their functional role, as reported in literature. We excluded SNPs with a call rate < 95% or deviation from Hardy–Weinberg equilibrium proportions (P<0.01) in the controls.

### DNA extraction, genotyping and genetic markers

DNA was extracted from frozen blood samples using DNeasy Blood & Tissue kit according to manufacturer’s instructions (QIAGEN). DNA was quantified and diluted for genotyping by real-time PCR with TaqMan probe assays (Life Technologies, EUA) (S2 Table). Assays consisted of 5 μL reactions containing 2.5 μL of TaqMan Universal PCR Master Mix No AmpErase UNG (Applied Biosystems), 1.375 μL of water, 0.125 μL of the TaqMan assay (primers and probes), and 1 μL of DNA template (10–40 ng). Genotyping was carried out in the StepOne Plus real-time PCR system (Applied Biosystems), by fluorescence-based allelic discrimination following standard cycling conditions.

We used a panel of 46 ancestry-informative autosomal Indels (AIM-Indels) that were genotyped in a single multiplex PCR followed by capillary electrophoresis, as described by Pereira and coauthors (2012) [26], using an ABI 3500 Genetic Analyzer (Life Technologies). Alleles were conferred in GeneMapper v.4.1 software (Life Technologies). Ancestry estimates for each of the three main population components (EUR, NAM and AFR) were determined using STRUCTURE v2.3.3 software [27] and the HGDP-CEPH reference sample panel [28].

### Statistical analysis

Linkage disequilibrium (LD) estimates for two SNPs in *NOD2* and two SNPs in *LRRK2* and deviations from Hardy–Weinberg equilibrium in the control group were performed using Haploview software, version 4.2 [29]. The estimated haplotypes was based on genotypes using an Expectation Maximization (EM) algorithm. Cases and controls were compared according to genotype, allele, and carrier frequencies, with and without adjustment for the variables: gender, age and ancestry (NAM and EUR, as continuous variables). Statistical analysis was performed using R software version 3.4.3 for Windows using “genetics” and “rms” packages (https://www.r-project.org) [30].

## Results

Patients and controls were ten years of age or older. A total of 412 leprosy patients (284 males, 128 females) and 967 controls (526 males, 441 females) were recruited (Table 1). Mean age was significantly lower in control subjects than in cases (mean ± SD, 29.8 ± 9.94 vs. 43.3 ± 18.14 years; P <0.0001). Likewise, the proportion of males and females was different between the two groups (P <0.001), with significantly more males among leprosy patients (Table 1).

**Table 1:** General characteristics of leprosy patients and controls.

Variables	Patients n = 412	Controls n = 967
**Age****^a^**	43.3 ± 18.14	29.8 ± 9.94
**Gender**^**b**^		
Male	284 (68.9%)	526 (54.4%)
Female	128 (31.1%)	441 (45.6%)
**WHO classification**		
PB	133	-
MB	279	-

WHO, World Health Organization. Paucibacillary (PB); Multibacillary (MB)

Student’s *t*-test for age between patients and control subjects P < 0.0001; Chi-squared test for gender P < 0.0001; Data shown as mean ± standard deviation.

^a^Student’s t-test

^b^Chi-squared test

The analyzed population demonstrated typical ancestral characteristics of admixed populations for European, Native American and African ancestry (S1 Fig).

All SNPs in the control group were in Hardy–Weinberg equilibrium (HWE). The *PACRG/PRKN* SNP rs1040079 was excluded from the analysis because the call rate was <95%. There were no statistically significant differences between cases and controls for SNPs *PACRG/PRKN*, *IL10*, *TNF*, *TLR1*, *CCDC122/LACC1*, *IL6*, *LRRK2*, *TYK2* and *IL23R* in the three genetic models (genotypic, allelic and carriers) (Table 2).

**Table 2:** Association of Allele, Genotype and Carrier frequencies of candidate genes with Leprosy.

SNP	GenotypeN (%)	ControlsN (%)	LeprosyN (%)	OR(95% CI)	*P-value*	Adjusted OR^a^(95% CI)	*P-value*
**rs8057341** ***NOD2***	AA	182 (19)	52 (13)	**0.56 (0.39–0.80)**	**0.0017**	**0.56 (0.37–0.84)**	**0.0052**
	GA	446 (47)	186 (46)	0.82 (0.64–1.05)	0.1220	0.85 (0.63–1.13)	0.2547
	GG^b^	328 (34)	167 (41)				
	Total	956	406				
**Allele**	A	0.42	0.36	**0.76 (0.60–0.96)**	**0.0243**	**0.76 (0.58–1.00**)	**0.0529**
	G^b^	0.58	0.64				
**Carriers**	AA/GA	628 (66)	239 (59)	**0.74 (0.59–0.95)**	**0.0153**	**0.76 (0.58–1.00)**	**0.0512**
**HWE**		0.1886					
**rs751271** ***NOD2***	TT	203 (21.0)	61 (15.0)	**0.63 (0.44–0.89)**	**0.0093**	0.67 (0.44–1.01)	0.0561
	GT	468 (49.0)	208 (51.0)	0.93 (0.71–1.20)	0.5738	0.93 (0.68–1.27)	0.6690
	GG^b^	286 (30.0)	137 (34.0)				
	Total	957	406				
**Allele**	T	0.46	0.41	0.81 (0.64–1.03)	0.0879	0.84 (0.63–1.10)	0.2092
	G^b^	0.54	0.59				
**Carriers**	TT/GT	671 (70.0)	269 (66.0)	0.84 (0.65–1.07)	0.1593	0.85 (0.64–1.15)	0.2976
**HWE**		0.7007					
**rs2430561** ***IFNG***	TT	55 (6.0)	29 (7.0)	1.39 (0.87–2.25)	0.1713	1.67 (0.97–2.89)	0.0641
	AT	341 (35.0)	164 (40.0)	1.27 (0.99–1.62)	0.0531	**1.40 (1.06–1.85)**	**0.0184**
	AA	566 (59.0)	214 (53.0)				
	Total	962	407				
**Allele**	T	0.23	0.27	1.22 (0.94–1.59)	0.1326	1.34 (0.99–1.81)	0.0584
	A^b^	0.77	0.73				
**Carriers**	TT/AT	396 (41)	193 (47)	1.29 (1.02–1.63)	0.0328	**1.44 (1.10–1.88)**	**0.0083**
**HWE**		0.7681					
**rs9356058** ***PACRG/*** ***PRKN***	CC	89 (9)	43 (11)	1.32 (0.88–1.98)	0.1764	1.36 (0.86–2.15)	0.1878
	TC	382 (41)	193 (48)	**1.38 (1.08–1.77)**	**0.0101**	1.27 (0.96–1.69)	0.0880
	TT^b^	465 (50)	170 (42)				
	Total	936	406				
**Allele**	T	0.70	0.66	0.81 (0.64–1.04)	0.1069	0.83 (0.63–1.10)	0.2011
	C^b^	0.30	0.34				
**Carriers**	CC/TC	471 (50)	236 (58)	**1.37 (1.08–1.73)**	**0.0086**	1.29 (0.99–1.68)	0.0603
**HWE**		0.4600					
**rs1800871** ***IL10***	AA	143 (15.0)	60 (15.0)	1.05 (0.73–1.50)	0.7925	0.93 (0.62–1.39)	0.7317
	GA	443 (46.0)	196 (48.0)	1.11 (0.86–1.42)	0.4351	0.94 (0.70–1.25)	0.6551
	GG^b^	375 (39.0)	150 (37.0)				
	Total	961	406				
**Allele**	A	0.38	0.39	1.04 (0.82–1.32)	0.7315	0.96 (0.73–1.26)	0.7599
	G^b^	0.62	0.61				
**Carriers**	AA/AG	586 (61.0)	256 (63.0)	1.09 (0.86–1.39)	0.4709	0.93 (0.71–1.23)	0.6303
**HWE**		0.5605					
**rs1800629** ***TNF***	AA	7 (1.0)	1 (0.0)	0.33 (0.04–2.66)	0.2954	0.14 (0.01–1.79)	0.1291
	GA	135 (14.0)	50 (12.0)	0.84 (0.60–1.20)	0.3431	0.78 (0.53–1.16)	0.2226
	GG^b^	824 (85.0)	361(88.0)				
	Total	966	412				
**Allele**	A	0.08	0.06	0.81 (0.51–1.28)	0.3604	0.74 (0.43–1.25	0.2559
	G^b^	0.92	0.94				
**Carriers**	AA/GA	142 (15.0)	51 (12.0)	0.82 (0.58–1.15)	0.2562	0.75 (0.51–1.11)	0.1536
**HWE**		0.7331					
**rs4833095** ***TLR1***	TT	213 (22.0)	102 (25.0)	1.21 (0.87–1.68)	0.2479	1.19 (0.77–1.62)	0.5553
	CT	482 (50.0)	207(50.0)	1.09 (0.82–1.44)	0.5542	1.00 (0.73–1.38)	0.9952
	CC^b^	261 (27.0)	103 (25.0)				
	Total	956	412				
**Allele**	T	0.47	0.50	1.10 (0.87–1.39)	0.4173	1.06 (0.81–1.37)	0,6841
	C^b^	0.53	0.50				
**Carriers**	TT/CT	695 (72.0)	30 (75.0)	1.13 (0.86–1.47)	0.3770	1.04 (0.77–1.40	0.8111
**HWE**		0.7851					
**rs2069845** ***IL6***	GG	94 (10.0)	38 (9.0)	1.03 (0.68–1.55)	O.8994	1.01 (0.63–1.60)	0.9751
	AG	400 (41.0)	182 (45.0)	1.16 (0.91–1.48)	0.2448	1.11 (0.84–1.47)	0.4676
	AA^b^	470 (49.0)	185 (46.0)				
	Total	964	405				
**Allele**	G	0.30	0.32	1.06 (0.83–1.37)	0.6208	1.04 (0.78–1.38)	0.7806
	A^b^	0.70	0.68				
**Carriers**	GG/AG	494 (51.0)	220 (54.0)	1.13 (0.90–1.43)	0.2985	1.09 (0.83–1.42)	0.5284
**HWE**		0.5600					
**rs4942254** ***CCDC122/*** ***LACC1***	CC	126 (13.0)	55 (14.0)	1.09 (0.75–1.57)	0.6548	1.10 (0.72–1.66)	0.6682
	CT	412 (44.0)	187(47.0)	1.13 (0.88–1.45)	0.3412	1.21 (0.91–1.62)	0.1938
	TT^b^	396 (42.0)	159 (40.0)				
	Total	934	401				
**Allele**	C	0.36	0.37	1.07 (0.84–1.36)	0.6040	1.09 (0.82–1.44)	0.5486
	T^b^	0.64	0.63				
**Carriers**	CC/CT	538 (57.0)	242 (61.0)	1.12 (0.88–1.42)	0.3505	1.18 (0.90–1.56)	0.2276
**HWE**		0.2849					
**rs7298930** ***LRRK2***	AA	125 (13.0)	52 (13.0)	1.00 (0.69–1.46)	0.9830	1.07 (0.70–1.63)	0.7589
	AC	418 (45.0)	195 (48.0)	1.13 (0.88–1.45)	0.3521	1.14 (0.86–1.52)	0.3564
	CC^b^	391 (42.0)	162 (40.0)				
	Total	934	409				
**Allele**	A	0.36	0.37	1.03 (0.81–1.32)	0.7807	1.06 (0.81–1.40)	0.6604
	C^b^	0.64	0.63				
**Carriers**	AA/AC	543 (58.0)	247 (61.0)	1.10 (0.87–1.39)	0.4399	1.13 (0.86–1.47)	0.3863
**HWE**		0.471					
**rs3761863** ***LRRK2***	TT	218 (23.0)	76 (20.0)	0.78 (0.55–1.09)	0.1412	0.90 (0.61–1.32)	0.5856
	CT	457 (48.0)	189 (49.0)	0.92 (0.70–1.21)	0.5546	0.97 (0.71–1.33)	0.8690
	CC^b^	274 (29.0)	123 (32.0)				
	Total	949	388				
**Allele**	T	0.47	0.44	0.88 (0.70–1.12)	0.3012	0.95 (0.72–1.24)	0.7067
	C^b^	0.53	0.56				
**Carriers**	TT/CT	675 (71.0)	265 (69.0)	0.87 (0.68–1.13)	0.3044	0.95 (0.71–1.27)	0.7352
**HWE**		0.3334					
**rs55882956** ***TYK2***	AG	2 (0.21)	2 (0.49)	2.36 (0.33 16.8)	0.3903	4.71 (0.61–36.3)	0.1366
	GG^b^	960 (99.79)	406 (99.51)				
	Total	962	408				
**Allele**	A	0.001	0.002	2.36 (0.15–37.8)	0.5439	4.69 (0.26–83.5)	0.2932
	G^b^	0.999	0.998				
**Carriers**	AA/AG	2 (0.21)	2 (0.49)	2.36 (0.33–6.84)	0.3903	4.71 (0.61–36.3)	0.1366
**HWE**		1					
**rs76418789** ***IL23R***	AG	2 (0.21)	1 (0.25)	1.17 (0.11–12.9)	0.8977	0.95 (0.01–60.5)	0.9804
	GG^b^	953 (99.79)	407 (99.75)				
	Total	955	408				
**Allele**	A	0.001	0.001	1.17 (0.04–34.9)	0.9276	0.95 (0.0–336.9)	0.9862
	G^b^	0.999	0.999				
**Carriers**	AA/AG	2 (0.21)	1 (0.25)	1.17 (0.11–12.9)	0.8977	0.95 (0.01–60.5)	0.9804
**HWE**		1					

Abbreviations: HWE, Hardy–Weinberg equilibrium; OR, odds ratio; SNP, single-nucleotide polymorphism. Bold values express statistically significant results.

^a^Results of logistic regression analyses adjusted for the covariates: gender, age and ancestry.

^b^Baseline. 95% CI, 95% confidence interval

The statistical power of the sample size from the present study was also evaluated considering the minor allele frequency (MAF) obtained from each of the SNPs and the Odds Ratio (OR) association effects ranging from 1.5 up to 2.5. For most of the SNPs the assessment of power attained 0.80 for the tested values, except for *TNF* SNP that was lower (0.52). The *TYK2* and *IL23R* SNPs had a rare frequency that resulted in power of less than 10%. Since no statistically significant associations were found for clinical forms comparison (PB vs MB and Controls vs MB) we did not include this power analysis.

The genotype, allele and carrier frequencies of *NOD2* rs8057341 confirmed association with protection from leprosy. The rs8057341 polymorphism was identified as significant for the AA genotype (OR = 0.56; 95% CI, 0.37–0.84; P = 0.005) and borderline for the A allele (OR = 0.76; 95% CI, 0.58–1.00; P = 0.053) and AA/GA carriers (OR = 0.76; 95% CI, 0.58–1.00; P = 0.051). The rs751271 polymorphism was identified as borderline for the TT genotype (OR = 0.67; 95% CI, 0.44–1.01; P = 0.056) (Table 2). High LD (r2 = 0.83) revealed dependent association for these two SNPs. Analysis of *NOD2* SNPs haplotypes (rs751271-rs8057341) were statistically significant for the T-A combination (OR = 0.79; 95% CI, 0.64–0.97; P = 0.0226), T-G combination (OR = 1.91; 95% CI, 1.21–3.01; P = 0.0055) and G-A (OR = 3.88; 95% CI, 1.50–10.04; P = 0.0052) (S3 Table).

SNP rs2430561 in the *IFNG* gene was associated with disease susceptibility in AT heterozygotes (OR = 1.40; 95% CI, 1.06–1.85; P = 0.018) and TT/AT carriers (OR = 1.44; 95% CI, 1.10–1.88; P = 0.008) (Table 2).

SNPs in the *LRRK2* gene showed a weak LD (r^2^ = 0.18). Analysis of *LRRK2* SNPs haplotypes was not statistically significant. The C-T combination (rs7298930—rs3761863) was shown to be borderline (P = 0.07), but lost association when adjusted for co-variates (OR = 0.78; 95% CI, 0.58–1.06; P = 0.1095) (S4 Table). Also, we tested rs9356058 in the *PACCRG*/*PRKN* genes and observed association when TC/CC carriers were evaluated, however, this was not sustained following co-variate adjustment (OR = 1.29; 95% CI, 0.99–1.68; P = 0.0603) (Table 2).

## Discussion

Genetic association studies in population designs, such as case-control, can lead to spurious associations, especially in admixed populations [31], which may confer false-positive results and the risk of developing a disease [32,33]. In our study, to reduce the chance of bias, we performed data adjustment by age, gender as well as ancestry. The current profile of the genetic ancestry of the Amazonas population began to form during the Portuguese colonization in the mid-16th century [34] and continued with the exploration of natural resources, primarily rubber [35]. Construction of the Transamazon Highway and the establishment of an Industrial Park (Manaus Duty Free Zone) in the 1960s also contributed to a huge migratory flow of Brazilians from different regions of the country [36]. The populations analyzed in our study had similar ancestral contributions in both case and control groups, which is ideal in genetic association studies. Nevertheless, the data was highly impacted by age adjustment since cases and controls had very different mean ages.

We validated *NOD2* and *IFNG* associations with resistance and risk of leprosy, respectively, in the Amazon ethnic admixed population. The *NOD2* gene encodes an intracellular sensing molecule, which, along with *NOD1*, recruits ATG16L1 to the plasma membrane to initiate autophagy of bacteria entering the host cell [37]. Similarly, Sales-Marques and coauthors (2014) [7], in a meta-analysis with population samples from different Brazilian regions combining case-control and family-based studies, confirmed that the A allele of *NOD2* (rs8057341) is a genetic resistance factor for leprosy. On the other hand, in the first GWAS in the Chinese population [4], the G allele (MAF = 0.22) of SNP rs8057341 was associated with disease risk. However, two other studies failed to validate this SNP in three populations: Indian, African and Vietnamese [5,38].

The SNP rs9356058 in *PACRG/PRKN*, for the T allele, points to protection, although statistical significance did not reach the threshold level. Although the specific function of the *PACRG* gene has not been elucidated [9], together with *PRKN*, they share a regulatory region and participate in the proteolytic system mediated by ubiquitin for the clearance of damaged biomolecules (lipids and proteins) and organelles [9,39]. The *PRKN* gene encodes the Parkin protein, an E3 Ubiquitin Ligase, the last sequential enzyme in the ubiquitination process [40]. Recently, the relationship of this protein with the innate immune response of the host, known as xenophagy, which is the degradation of intracellular pathogens [41], as described for *M*. *tuberculosis* [42], Chlamydia [43] and *M*. *leprae* [44] has been identified. Polymorphic variants in the *PRKN* gene were initially associated with the autosomal recessive form of Parkinson's disease (AR-JP) [45]. In 2004, Mira and coauthors [3] identified polymorphisms in the promoter region shared by *PACRG/PRKN* genes associated with leprosy *per se* in Southern Brazilians and Vietnamese. In this same study, the common T allele of rs9356058 was associated with the risk of leprosy. Later, Alter and coauthors confirmed that this variant (T allele of rs9356058) is a risk factor for the disease in the Vietnamese and Indian populations [46]. In this study, *PRKN* analysis suggested that genetic association is higher in children/youths, when data is stratified for age. Since our population was older (40 years old) and the mean age of the control group varied, statistical adjustments might explain different results. The lack of consensus among ours and previous studies for the *PRKN* region is probably due to distinct patterns of LD in the analyzed populations, even within the same country, leading to differences in allele and haplotype frequencies of the studied SNPs, as previously observed for lymphotoxin-alpha [47]. It is likely that NAM ancestry could explain this pattern. Noteworthy, studies in Indian and Chinese populations failed to replicate the association of these *PRKN* polymorphisms with leprosy [48, 49].

Interferon gamma (*IFNG*) is one of the most significant cytokines involved in the protective immune response against mycobacterial infection and is secreted mainly by CD4+ Th1, CD8+ T cytotoxic and natural killer cells [50]. In synergy with TNF, it activates microbicide effector mechanisms in human macrophages [51]. IFNγ expression may be influenced by the polymorphism present in the first intron of IFNG +874 T>A rs2430561, probably since this locus coincides with the binding site of the NF-kB transcription factor [52]. This polymorphism has been associated in meta-analysis studies with leprosy [24], tuberculosis [53] and Hepatitis [50]. Our results indicated T allele carriers have an increased risk of leprosy (P = 0.0083). A new meta-analysis would indicate a consensus estimate for this SNP. In studies that show an association, the presence of the T allele correlates with high IFNG expression and increased resistance to infection whereas the A allele correlates with low expression [54–56]. On the other hand, in vitro clinical trials showed that interferon levels were not statistically different between T carriers and AA genotype, in the presence of *M*. *leprae* antigens [57].

We did not validate associations for SNPs in *TLR1*, *TNF*, *PACRG/PRKN*, *IL10*, *CCDC122/LACC1*, *IL6*, *LRRK2*, *IL23R*, and *TYK2* genes. Nevertheless, some of these SNPs, such as rs3761863 in *LRRK2* had been associated with a type-1 reaction, which is considered an endophenotype of leprosy [58]. These SNPs could possibly play a role in the uncontrolled inflammatory phenotype throughout the natural course of the disease. Recently, rare *LRRK2* SNPs were confirmed as associated with either leprosy type-1 reactions or Parkinson´s Disease [59]. Hence, we also tested SNPs from *TYK2* and *IL23R*, both low-frequency variants that were described in the Chinese population [6]. In the present Amazon population sample, these variants were rare leading to a low detection power of association with leprosy. Although significance was not found, *TYK2* heterozygote frequency was two times higher among patients. However, we cannot rule out the possibility of other rare or common variants of *IL23R* or *TYK2* being related to leprosy susceptibility in the Amazon population.

The few common SNPs that showed association with leprosy and the modest odds ratios values presented demonstrate the difficulty of unraveling the major genes involved in leprosy. Genome-wide association studies and exome analysis could possibly improve the ability to describe novel rare SNPs and call on a combination of different genotypes to explain one complex phenotype. It is likely that this can help define genetic variants and understand their role in the pathophysiology of leprosy, contributing to either diagnosis or treatment [39].

## Supporting information

S1 Fig Individual ancestry estimates obtained for the HGDP-CEPH reference samples and individuals tested from CASES (Leprosy) and CONTROLS (Endemic Control) STUDY using 46 AIM-Indels (AFR: African; EUR: European; NAM: Native American). (TIF)

S1 Table Summary of candidate genes of immune response in the case-controls studies in Leprosy. (DOC)

S2 Table Genotyping assays used for allelic discrimination. (DOC)

S3 Table Haplotypes of the intron region of NOD2 present in the study population. (DOC)

S4 Table Haplotypes of the LRRK2 present in the study population. (DOC)
