# Correction: Association of *NOD2* and *IFNG* single nucleotide polymorphisms with leprosy in the Amazon ethnic admixed population

**DOI:** 10.1371/journal.pntd.0011228

**Published:** 2023-03-24

**Authors:** André Luiz Leturiondo, Ariani Batista Noronha, Carla Yael Ribeiro Mendonça, Cynthia de Oliveira Ferreira, Lucia Elena Alvarado-Arnez, Fernanda Saloum de Neves Manta, Ohanna Cavalcanti de Lima Bezerra, Elizeu Fagundes de Carvalho, Milton Ozório Moraes, Fabíola da Costa Rodrigues, Carolina Talhari

The Funding statement is incomplete. The complete statement should read:

This study was supported by FAPEAM (Fundação de Amparo a Pesquisa do estado do Amazonas, Brazil) through: Programa Pesquisa para o SUS: Gestão Compartilhada em Saúde—PPSUS, Chamada Pública FAPEAM/SUSAM-SES-AM/MS/CNPq n.º 001/2013—CONSELHO DIRETOR–DECISÃO n.º 287/2013 (http://www.fapeam.am.gov.br/wp-content/uploads/2013/05/Chamada-Publica-001_2013-PPSUS-Decisao-CD-287-2013.pdf); Programa de Pós-Graduação em Medicina Tropical da UEA/FMT-HVD; Programa de Apoio à Publicação de Artigos Cientificos–PAPAC, Edital n.º 005/2019 (http://www.fapeam.am.gov.br/wp-content/uploads/2019/08/Decisao-CD-204-2019-Proc.-506.2019-Homologacao-do-Resultado-PAPAC-Edital-nA--005_2019.pdf); and Programa de Apoio à Consolidação das Instituições Estaduais de Ensino e/ou pesquisa–PRO-ESTADO n.º 423–2018.
