# Supplementary figures and images for: Association of NOD2 and IFNG single nucleotide polymorphisms with leprosy in the Amazon ethnic admixed population
Source: PLoS Negl Trop Dis. 2020 May 20;14(5):e0008247. doi: 10.1371/journal.pntd.0008247 (PMC7239438; doi:10.1371/journal.pntd.0008247)

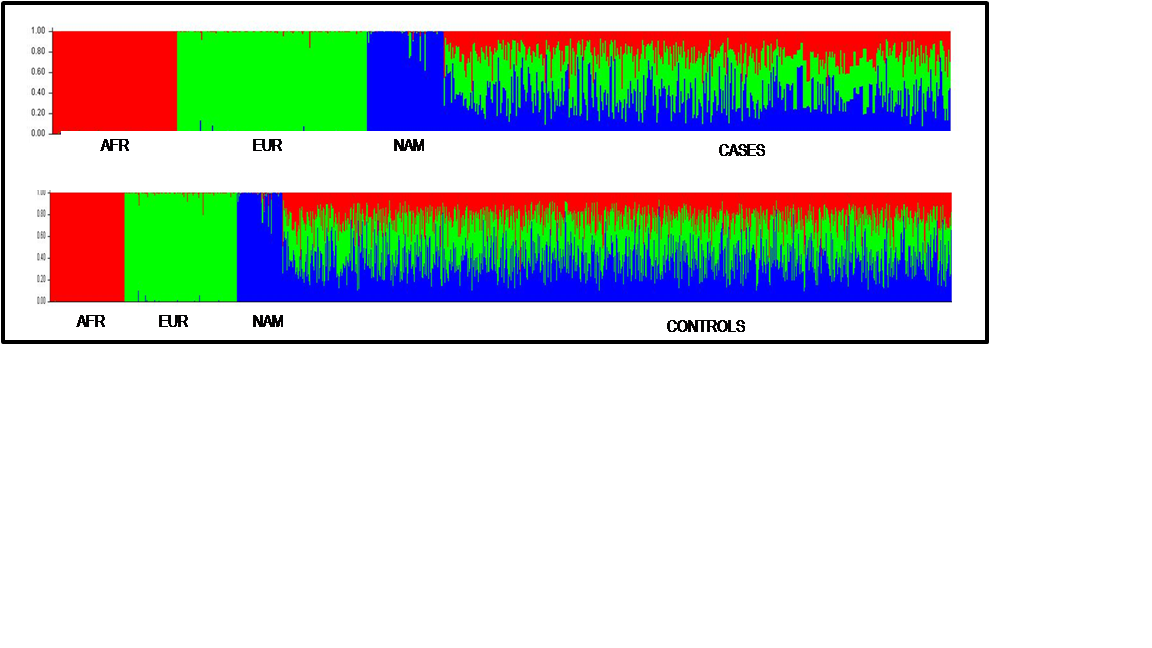

Supplement: S1 Fig — (TIF) [file pntd.0008247.s001.tif]
