# Supplementary material for: Association of NOD2 and IFNG single nucleotide polymorphisms with leprosy in the Amazon ethnic admixed population
Source: PLoS Negl Trop Dis. 2020 May 20;14(5):e0008247. doi: 10.1371/journal.pntd.0008247 (PMC7239438; doi:10.1371/journal.pntd.0008247)
Supplement: S1 Table — (DOC) [file pntd.0008247.s002.doc]

Supplementary Table 1. Summary of candidate genes of immune response in the case-controls studies in Leprosy

| **Gene** | **SNPs** | **Association**  **Yes No** | **Outcome** | **Population** | **References** |
| --- | --- | --- | --- | --- | --- |
| ***NOD2*** | **rs8057341** | **Yes**  **Yes**  **No**  **No** | **Leprosy**  **Leprosy**  **Leprosy**  **Leprosy** | **Chinese**  **Brazilian**  **Vietnamese**  **Indian */*African** | **[4]**  **[7]**  **[38]**  **[5]** |
| ***NOD2*** | **rs751271** | **Yes**  **Yes** | **Reaction**  **Leprosy** | **Brazilian**  **Nepalese** | **[14]**  **[15]** |
| ***TLR1*** | **rs4833095** | **Yes**  **Yes**  **Yes** | **Leprosy**  **Leprosy**  **Reaction** | **Brazilian**  **Bangladesh**  **Bangladesh** | **[10]**  **[16]**  **[16]** |
| ***TNF*** | **rs1800629** | **Yes** | **Leprosy** | **Brazilian** | **[11]** |
| ***IL10*** | **rs1800871** | **Yes**  **Yes** | **Leprosy**  **PB** | **Brazilian/Indian/**  **Chinese/Malawian**  **Brazilian** | **[13]**  **[17]** |
| ***IFNG*** | **rs2430561** | **Yes**  **Yes** | **Leprosy**  **PB** | **Brazilian */*Chinese**  **Brazilian** | **[24]**  **[18]** |
| ***PACRG /***  ***PRKN*** | **rs9356058** | **Yes**  **Yes**  **Yes**  **Yes**  **No**  **No**  **No** | **Leprosy**  **Leprosy**  **Leprosy**  **Leprosy**  **Leprosy**  **Leprosy**  **Leprosy** | **Brazilian**  **Vietnamese**  **Vietnamese**  **Indian**  **Indian**  **Chinese**  **Indian** | **[3]**  **[3]**  **[46]**  **[46]**  **[9]**  **[49]**  **[48]** |
| ***PACRG /***  ***PRKN*** | **rs1040079** | **Yes**  **Yes**  **Yes**  **No**  **No**  **No**  **No** | **Leprosy**  **Leprosy**  **Leprosy**  **Leprosy**  **Leprosy**  **Leprosy**  **Leprosy** | **Brazilian**  **Vietnamese**  **Vietnamese**  **Indian**  **Indian**  **Chinese**  **Indian** | **[3]**  **[3]**  **[46]**  **[46]**  **[9]**  **[49]**  **[48]** |
| ***CDC122/ LACC1*** | **rs4942254** | **Yes** | **Leprosy** | **Brazilian** | **[7]** |
| ***IL6*** | **rs2069845** | **Yes** | **Reaction** | **Brazilian** | **[14]** |
| ***LRRK2*** | **rs7298930** | **Yes** | **Leprosy** | **Chinese** | **[8]** |
| ***LRRK2*** | **rs3761863** | **Yes** | **Leprosy** | **Chinese** | **[8]** |
| ***IL23R*** | **rs76418789** | **Yes** | **Leprosy** | **Chinese** | **[6]** |
| ***TYK2*** | **rs55882956** | **Yes** | **Leprosy** | **Chinese** | **[6]** |
