# Supplementary material for: Association of NOD2 and IFNG single nucleotide polymorphisms with leprosy in the Amazon ethnic admixed population
Source: PLoS Negl Trop Dis. 2020 May 20;14(5):e0008247. doi: 10.1371/journal.pntd.0008247 (PMC7239438; doi:10.1371/journal.pntd.0008247)
Supplement: S2 Table — (DOC) [file pntd.0008247.s003.doc]

Supplementary Table 2. Genotyping assays used for allelic discrimination

| **SNP** | **Gene** | **M/m** | **MAF^1^** | **Functional Consequence** | [**TaqMan® SNP Genotyping Assays**](https://www.thermofisher.com/us/en/home/life-science/pcr/real-time-pcr/real-time-pcr-assays/snp-genotyping-taqman-assays.html) |
| --- | --- | --- | --- | --- | --- |
| rs8057341 | *NOD2* | G/A | common | Intron variant | C_3017466_10 |
| rs751271 | *NOD2* | T/G | common | Intron variant | C_1384442_10 |
| rs4833095 | *TLR1* | C/T | common | missense | [**C_44103606_10**](https://www.thermofisher.com/order/genome-database/details/genotyping/C__44103606_10?CID=&ICID=&subtype=) |
| rs1800629 | *TNF* | G/A | common | upstream variant | C_7514879_10 |
| rs1800871 | *IL-10* | G/A | common | upstream variant | C_1747362_10 |
| rs2430561 | *IFNG* | T/A | common | Intron variant | AH20TEB |
| rs9356058 | *PACRG/*  *PRKN* | T/C | common | Intron variant | C_179028_10 |
| rs1040079 | *PACRG/*  *PRKN* | A/G | common | Intron variant | C_1575898_10 |
| rs4942254 | *CCDC122/*  *LACC1* | T/C | common | Intron variant upstream variant | C_30513886_10 |
| rs2069845 | *IL-6* | A/G | common | Intron variant | C_1839699_10 |
| rs7298930 | *LRRK2* | C/A | common | Intron variant | C_2069047_10 |
| rs3761863 | *LRRK2* | C/T | common | missense | C_3215842_10 |
| rs76418789 | *IL-23R* | G/A | rare | missense | C_104780482_10 |
| rs55882956 | *TYK2* | G/A | rare | missense | C_89470466_10 |

SNP- Single Nucleotide Polymorphism; M/m, Major/minor allele; *NOD2*, nucleotide binding oligomerization domain containing 2; *TLR1*, Toll like receptor 1; *TNF*, tumor necrosis factor; *IL10*, interleukin 10; *IFNG*, Interferon gamma; *PACRG*, parkin coregulated; *PRKN*, parkin RBR E3 ubiquitin protein ligase; *CCDC122*, coiled-coil domain containing 122 gene; *LACC1*, Laccase multicopper oxidoreductase domain containing 1 gene; *IL6*, interleukin 6; *LRRK2*, Leucine rich repeat kinase 2; *IL23R*, Interleukin 23 receptor; *TYK2*, Tyrosine kinase 2.

common: minor allele frequency ≥ 5% in patients; rare: 1% < minor allele frequency in patients.

^1^MAF, Minor allele frequency, based in the 1000 Genomes Project Phase 3, in different populations such as Europeans, Mexican, Peruvian and Africans.
