# Supplementary material for: Association of NOD2 and IFNG single nucleotide polymorphisms with leprosy in the Amazon ethnic admixed population
Source: PLoS Negl Trop Dis. 2020 May 20;14(5):e0008247. doi: 10.1371/journal.pntd.0008247 (PMC7239438; doi:10.1371/journal.pntd.0008247)
Supplement: S3 Table — (DOC) [file pntd.0008247.s004.doc]

| **Haplotype** | **NOD2**  **rs751271** | **NOD2**  **rs8057341** | **Case (%)** | **Control (%)** | **OR (95% CI)** | **Adjusted OR (95% CI)^b^** |
| --- | --- | --- | --- | --- | --- | --- |
| **1** | **T** | **A** | **141 (34.2)** | **405 (41.9)** | **0.77 (0.65-0.92); P=0.0037** | **0.79 (0.64-0.97); P=0.0226** |
| **2** | **T** | **G** | **26 (6.4)** | **35 (3.6)** | **1.62 (1.10-2.37); P=0.0141** | **1.91 (1.21-3.01); P=0.0055** |
| **3** | **G** | **A** | **7 (1.7)** | **6 (0.6)** | **2.39 (1.09-5.21); P=0.0287** | **3.88 (1.50-10.04); P=0.0052** |
| **4 ^a^** | **G** | **G** | **238 (57.7)** | **521 (53.9)** |  |  |

Supplementary Table 3: Haplotypes of the intron region of NOD2 present in the study population.

^a^Haplo.base; ^b^Results of logistic regression analyses adjusted for the covariates: gender, age and ancestry; 95% CI, 95% confidence interval
