# Supplementary material for: Association of NOD2 and IFNG single nucleotide polymorphisms with leprosy in the Amazon ethnic admixed population
Source: PLoS Negl Trop Dis. 2020 May 20;14(5):e0008247. doi: 10.1371/journal.pntd.0008247 (PMC7239438; doi:10.1371/journal.pntd.0008247)
Supplement: S4 Table — (DOC) [file pntd.0008247.s005.doc]

| **Haplotype** | **LRRK2**  **rs7298930** | **LRRK2**  **rs3761863** | **Case (%)** | **Control (%)** | **OR (95% CI)** | **Adjusted OR (95% CI)^b^** |
| --- | --- | --- | --- | --- | --- | --- |
| **1** | **A** | **C** | **39 (9.4)** | **84 (8.7)** | **1.03 (0.73-1.46); P=0.8630** | **0.96 (0.63-1.46); P=0.8502** |
| **2** | **A** | **T** | **112 (27.2)** | **261 (27)** | **0.96 (0.79-1.17); P=0.6933** | **1.02 (0.81-1.29); P=0.8652** |
| **3** | **C** | **T** | **69 (16.8)** | **194 (20.1)** | **0.79 (0.62-1.02); P=0.0743** | **0.78 (0.58-1.06); P=0.1095** |
| **4^a^** | **C** | **C** | **192 (46.6)** | **428 (44.2)** |  |  |

Supplementary Table 4: Haplotypes of the LRRK2 present in the study population.

^a^Haplo.base; ^b^Results of logistic regression analyses adjusted for the covariates: gender, age and ancestry; 95% CI, 95% confidence interval
